# A novel in vitro periodontal pocket model to evaluate the effect of root surface instrumentation on biofilm-epithelial cell interactions

**DOI:** 10.1007/s00784-022-04371-7

**Published:** 2022-01-19

**Authors:** Kiri N. Lang, Anton Sculean, Sigrun Eick, Alexandra Stähli

**Affiliations:** Department of Periodontology, School of Dental Medicine, University of Bern, Freiburgstrasse 7, 3010 Bern, Switzerland

**Keywords:** Biofilm-epithelial cell interactions, Periodontal pocket model, Root surface instrumentation

## Abstract

**Objective:**

To develop a novel in vitro periodontal pocket model for evaluating the effect of two different root surface instrumentation modalities on biofilm-epithelial cell interactions.

**Materials and methods:**

An artificial periodontal pocket model was created using an impression material. Dentin discs were prepared and incubated for 3.5 days with a biofilm consisting of 12 bacterial strains. Then, the discs were inserted into the pocket model and instrumented for 10 s or 10 strokes either with ultrasonics (US) or hand instruments (HI). Subsequently, a glass slide coated with epithelial cells was placed in close vicinity to the discs. After incubation of the pocket model in a 5% CO_2_ atmosphere for 6 h, residual bacteria of the biofilm as well as bacteria adhering to or invaded into epithelial cells were determined using colony-forming unit (cfu) counts and real-time PCR. Further, as a parameter of the pro-inflammatory cell response, interleukin (IL)-8 expression was determined by ELISA.

**Results:**

Compared to untreated control, HI reduced the cfu counts by 0.63 log10 (not significant) and US by 1.78 log10 (*p* = 0.005) with a significant difference between the treatment modalities favoring US (*p* = 0.048). By trend, lower detection levels of *Tannerella forsythia* were detected in the US group compared to HI. Concerning the interaction with epithelial cells, half of the control and the HI samples showed epithelial cells with attaching or invading bacteria, while US displayed bacteria only in two out of eight samples. In addition, US resulted in significantly lower IL-8 secretion by epithelial cells compared to the untreated control. Between HI and controls, no statistically significant difference in IL-8 secretion was found.

**Conclusion:**

This newly developed in vitro model revealed in terms of biofilm-epithelial cell interaction after root surface instrumentation that compared to hand curettes, ultrasonic instrumentation appeared to be more effective in removing bacterial biofilm and in decreasing the inflammatory response of epithelium to biofilm.

**Clinical relevance:**

Ultrasonic instrumentation might be more advantageous to reduce cellular inflammatory response than hand instruments.

## Introduction

Periodontitis is an opportunistic infectious disease caused by oral bacteria and their interplay with the intricacy of the host’s immune and inflammatory response. Its primary features include the inflammatory-driven destruction of periodontal tissues resulting in periodontal pocketing, bleeding on probing, clinical attachment, and bone loss. The elimination of the biofilm is still the major goal of periodontal therapy (Löe et al. 1965; Salvi et al. 2012) and includes mechanical instrumentation—either manually or with ultrasonic instruments—of the exposed root surfaces. An alternative approach would be to influence the inflammatory response itself which is predominantly, if not entirely, responsible for tissue destruction. In the majority of cases, however, mechanical instrumentation is sufficient to induce healing of the periodontal tissues.

There has been a long lasting discussion on whether power-driven instruments (sonic and ultrasonic scalers) are to be preferred over hand instruments [8]. The consensus is that there is no difference in terms of clinical results between the two treatment modalities [14, 35] but that—in order to achieve an optimum—the combination of hand and ultrasonic instrumentation may be preferred [11]. Regarding substance loss, the least loss was noted for ultrasonic, then sonic, and finally hand instruments [29]. Thereby, hand curettes showed a mean substance loss per stroke of 6.8 µm when low forces were applied and 20.6 µm for high forces [37]. Ultrasonic instrumentation yielded smoother surfaces than the curette [7]. Regarding fibroblast survival and proliferation, no differences were found between hand curettes or ultrasonic systems [19]. In vitro models to investigate cell-biofilm interactions and therapeutic modalities are important tools in understanding not only the pathogenesis of periodontal disease but also its therapy. New experimental models could shed light on differences between ultrasonic and hand instrumentation in terms of microbiological and immune-inflammatory parameters.

In a previous study of our group, a similar in vitro pocket model was used that demonstrated considerable differences in biofilm removal and its reformation, surface alterations, and attachment of periodontal ligament fibroblasts when applying different non-surgical treatment modalities [12]. Hand curettes demonstrated the least biofilm reduction, while the use of an ultrasonic scaler resulted in the highest biofilm reduction. Surfaces after ultrasonication, in return, attracted the highest counts of PDL fibroblasts. Another group simulated the host–pathogen interplay in an in vitro multispecies biofilm model with *Streptococcus mitis*, *Fusobacterium nucleatum*, *Porphyromonas gingivalis*, and *Aggregatibacter actinomycetemcomitans* and oral epithelial cells to evaluate the effect of naturally derived polyphenol resveratrol and chlorhexidine [25]. Both compounds produced a downregulation of IL-8. An even more complex in vitro model mimicking the periodontal pocket challenged gingival epithelial keratinocytes, gingival fibroblasts, and monocytic cells (Mono-Mac 6) in a 3D collagen sponge with an 11-species biofilm [2]. As it was demonstrated, the cell conglomerate suppressed selective biofilm species implying an overall antimicrobial effect of the cells. Of course, their secretion of pro-inflammatory cytokines (IL-1β, IL-2, IL-8, TNF-α) was significantly increased in the presence of the biofilm [2]. Another in vitro immune cell-gingival tissue-biofilm model suggests that gingival epithelium modulates the cytokine expression of immune cells and vice versa [4]. When gingival epithelium was co-cultured with monocytes, IL-8 was increasingly expressed in monocytes, while it was downregulated in gingival epithelium [4]. Increased expression of the antimicrobial cytokine CCL20 and the inflammatory cytokines CXCL8 (IL-8) and IL-6 was observed in a multi-layered gingival epithelium grown on a collagen hydrogel when exposed to different biofilms [5]. Interestingly, the inflammatory response was stronger when the tissue model was exposed to a commensal oral biofilm than to a gingivitis-associated one [5].

IL-8 is a major chemoattractant cytokine and activator of neutrophils in both health and disease. Tissues of patients with chronic periodontitis show higher levels of IL-8 which is expressed by various cell types at sites of inflammation [26]. Oral bacteria induce the expression of IL-8 in epithelial cells. Whether lipopolysaccharide (LPS), a key virulence factor of gram-negative bacteria, also triggers IL-8 expression in epithelial cells is unknown. Recent data suggest that gingival epithelial cells might increase their responsiveness to LPS during episodes of dysbiosis and inflammation [18].

These aforementioned models were established to investigate host-microbiome interactions. However, there is limited evidence of models integrating biofilm-cell interactions in combination with therapeutic tools. As ultrasonic instrumentation uses water irrigation and is associated with less cementum removal, we hypothesized that manual and ultrasonic instrumentation may differ in the induced inflammatory response. Therefore, we sought to establish an in vitro biofilm-epithelial cell pocket model and to investigate how two different instrumentation modalities, hand and ultrasonic instrumentation, influence the biofilm removal and the interaction with epithelial cells.

## Materials and methods

### Ethical approval

Dentin discs were prepared from extracted human molar teeth that had been extracted for periodontal reasons during regular treatment from patients having given written informed consent. As teeth were anonymously collected, no ethical approval was required according to the guidelines set by the Ethics Committee of the University of Bern.

#### Preparation of specimens and pocket model

**Fig. 1:**
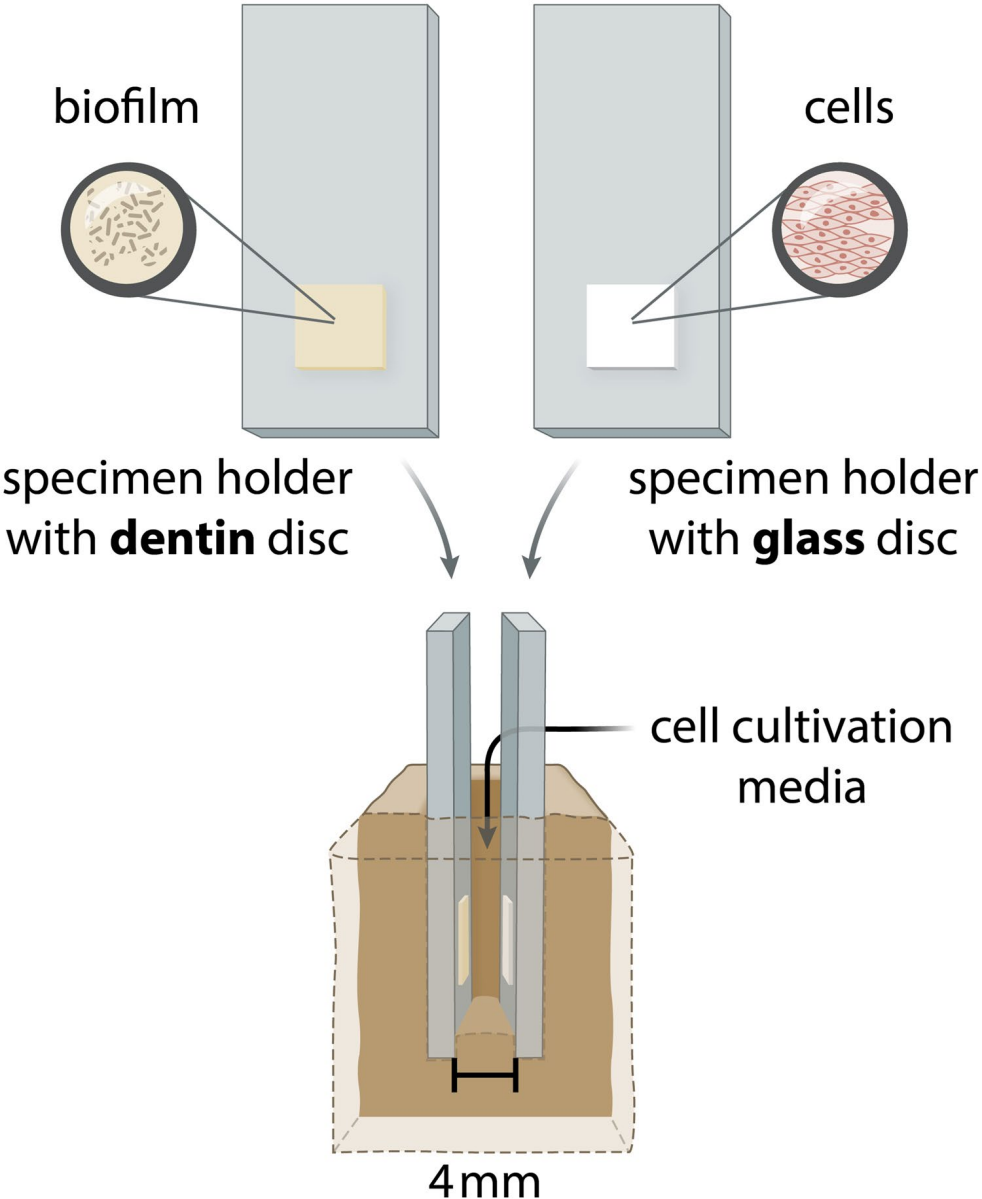
Illustration of the pocket model used for the experiments

Out of the extracted teeth, dentin discs with the size of 5 × 5 × 1 mm were made by using a diamond burr and grinding turntables. Next, they were adhesively attached to plastic specimen holders with a dentin adhesive system (Syntac, Ivoclar Vivadent, Schaan, Lichtenstein). In order to expose the dentin discs to gingival epithelial cells, a pocket model was created where the specimen holder with the dentin disc (and biofilm) and another one with a glass slide (with epithelial cells) could be put in close contact to each other with a distance of 4 mm. The pocket model was fabricated with a silicon dental impression material (Optosil, Kulzer GmbH, Hanau, Germany). The two specimen holders together with a spacer of 4-mm thickness were placed into the silicon mass and kept in situ during the hardening process. With this procedure, pockets of the same size were created. Pocket models were completely dipped in distilled water and autoclaved (Fig. 1).

#### Biofilm formation

The dentin discs on the plastic specimens were colonized with a biofilm consisting of 12 bacterial strains (*Streptococcus gordonii* ATCC 10,558, *Actinomyces naeslundii* ATCC 12,104, *Fusobacterium nucleatum* ATCC 25,586, *Campylobacter rectus* ATCC 33,238, *Eubacterium nodatum* ATCC 33,099, *Eikenella corrodens* ATCC 23,834, *Parvimonas micra* ATCC 33,270, *Filifactor alocis* ATCC 33,099, *Prevotella intermedia* ATCC 25,611, *Porphyromonas gingivalis* ATCC 33,277, *Tannerella forsythia* ATCC 43,037, *Treponema denticola* ATCC 35,405). Bacterial strains (except for *T. denticola*) were cultivated on Schaedler Agar plates (Oxoid Basingstoke, UK) with 5% sheep blood in an anaerobic atmosphere or with 5% CO_2_. *T. denticola* was maintained in mycoplasma broth (BD, Franklin Lakes, NJ) supplemented with niacinamide, spermine tetrahydrochloride, and cocarboxylase in anaerobic conditions.

Dentin specimens were covered with 1.5% bovine serum albumin for 15 min, before they were placed into tubes with nutrient broth (Wilkins-Chalgren broth with nicotinamide adenine dinucleotide and N-acetyl muramic acid) and bacteria. The tubes have been incubated in anaerobic conditions at 37 °C for 3.5 days. After 48 h, *P. gingivalis* ATCC 33,277, *T. forsythia* ATCC 43,037, and *T. denticola* ATCC 35,405 were added again to guarantee a colonization of these bacteria in biofilm.

#### Oral epithelial cells

Thin glass slides of a size of 5 mm × 5 mm were cut and fixed on a microscope slide. The glass slides were coated overnight with 0.01% poly-l-lysine solution (Sigma-Aldrich, Buchs, Switzerland). Then, telomerase-inactivated gingival keratinocyte (TIGK) cells (ATCC-CRL-3397) in cell cultivation media (Keratinocyte Growth Medium, KGM-Gold, Lonza, Basel, Switzerland) were added. Before instrumentation, cells were checked that they had grown to a confluent monolayer.

#### Instrumentation

After 3.5 days of biofilm formation, specimens with the dentin discs and the biofilm were transferred from the tube to the silicon form of the periodontal pocket model. Then, two different treatment modalities were applied. Dentin specimens were scaled either with 10 strokes at average working pressure using 11GC12 Gracey hand curettes (Gracey curettes, Deppeler SA, Rolle, Switzerland) or an ultrasonic device (W&H piezo scaler with W&H tips 2U) with water and power setting according to the manufacturer’s instruction for 10 s. All treatments were performed by an experienced periodontist (AS). A negative control with no applied treatment completed the groups.

#### Exposure of treated biofilm to epithelial cells

Biofilm-inoculated and then within the pocket model-treated dentin specimens were shortly taken out of the silicon model, dipped into PBS, and transferred back to the mold. Then, the specimen holder with the TIGK cells was placed in the pocket model with the cells facing the treated dentin specimens at close distance of 4 mm. Cell cultivation medium was added until both slides (with biofilm or TIGK cells) were covered. Thereafter, the periodontal pocket model with the treated biofilm and the TIGK cells has been incubated at 37 °C, 5% CO_2_, and 95% humidity for 6 h. Thereafter, supernatant was removed and kept frozen at − 80 °C until later assayed for IL-8 concentration. The specimens with the biofilm remains and with the epithelial cells were also taken out of the periodontal pocket model and processed further.

#### Analysis of biofilms on dentin specimen

Specimens with the dentin discs and the remained biofilm were shortly dipped into PBS. Thereafter, the biofilm was removed from the surface by intensive swabbing with a cotton swab which was given in a tube with PBS. After intensive mixing by pipetting and vortex, aliquots were spread on agar plates. Here, colony-forming units (cfu) were enumerated after an anaerobic incubation for 7 days. From another aliquot, DNA was extracted which was proceeded for quantification of *P. gingivalis*, *T. forsythia*, *T. denticola*, *P. intermedia*, *F. nucleatum*, and *C. rectus* counts by using real-time PCR as described before [9].

#### Analysis of bacteria attached and invasive to epithelial cells

Specimens with epithelial cells were shortly dipped into PBS and thereafter placed into ice-cold water for 15 min. Then, after intensive mixing, aliquots were proceeded as before. This method quantifies the bacteria attaching to epithelial cells or being already invasive to the cells.

#### Analysis of released interleukin-8

The cell culture medium exposed to the treated biofilm and epithelial cells was analyzed for its IL-8 concentration by using commercially available enzyme-linked immunosorbent assay (ELISA) kits (R&D Systems, Minnesota, MN, USA) according to the manufacturer’s instruction.

#### Statistical analysis

The final experiments were run in four series with each two specimen pairs (dentin, epithelium) per group. Statistical analysis was made by using SPSS 26.0 (IBM Corporation, New York, NY, USA). Groups were compared with the Kruskal–Wallis test followed by the Mann–Whitney test with Bonferroni correction. Significance was set at *p* < 0.05.

## Results

### Biofilm removal

**Fig. 2:**
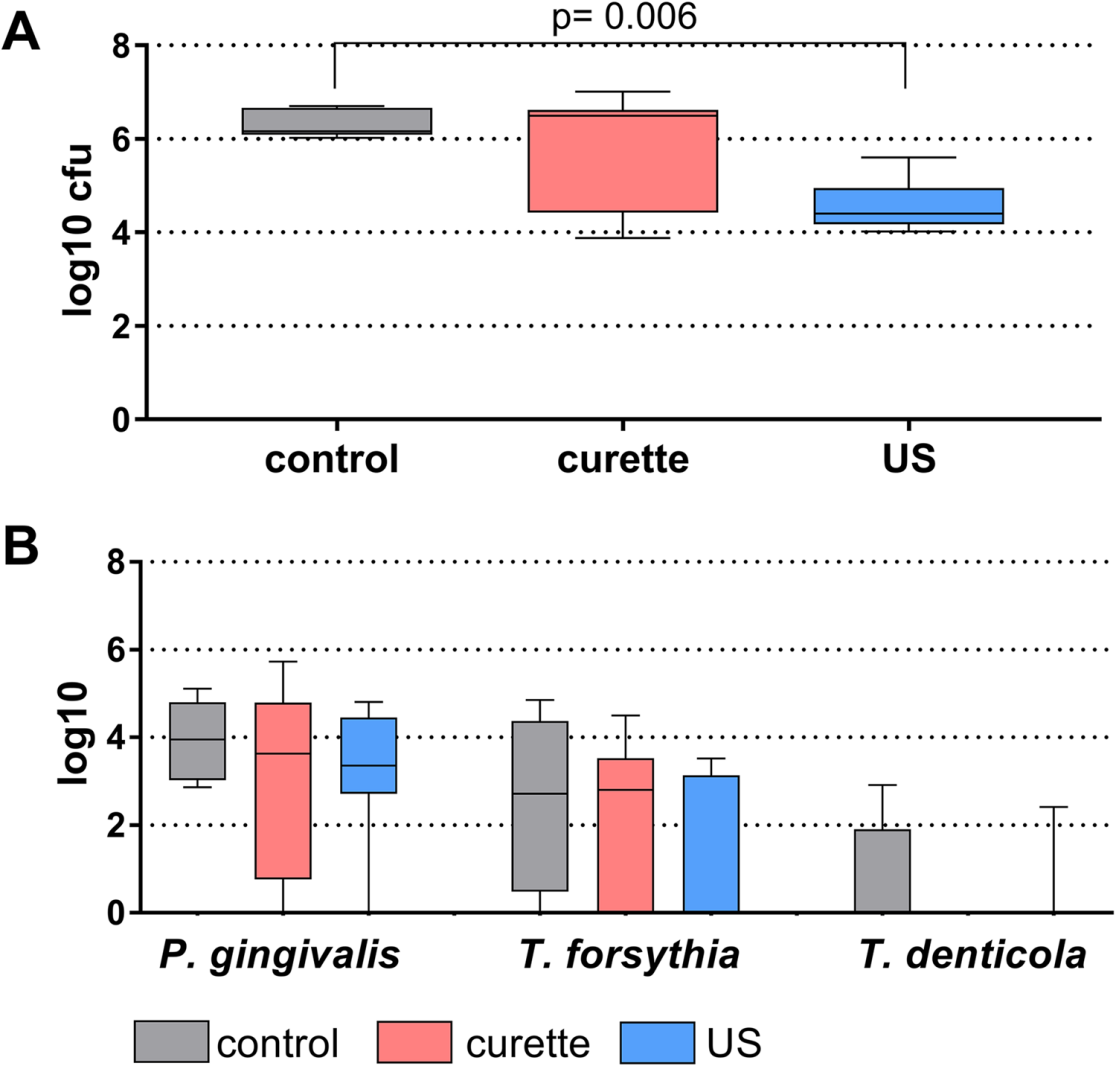
Median, quartiles, and range of total colony-forming units (cfu; **A**) and counts of selected bacterial species (determined by real-time PCR; **B**) of the remained biofilm on dentin discs 6 h after applying hand instrumentation (curette) and an ultrasonic scaler to biofilms on dentin discs

The cfu in biofilms 6 h after instrumentations differed statistically significantly (*p* = 0.004). The curettes reduced the counts by 0.63 log10 (not significant) and US by 1.78 log10 (*p* = 0.005) compared to the untreated control. In addition, there was a difference between the two treatment modalities in favor of US (*p* = 0.048; Fig. 2A).

Concerning the detection of single bacterial species by PCR 6 h after treatment, it was revealed that *P. gingivalis* was detected in all control biofilm samples and nearly in all of the test biofilm samples. *T. forsythia* was found in six and *T. denticola* in two of the eight control samples. Lower detection levels were obtained for both test groups although without any statistically significant difference when comparing the three groups. By trend, counts of *T. forsythia* were lower after US (Fig. 2B).

### Interaction with epithelial cells

**Fig. 3:**
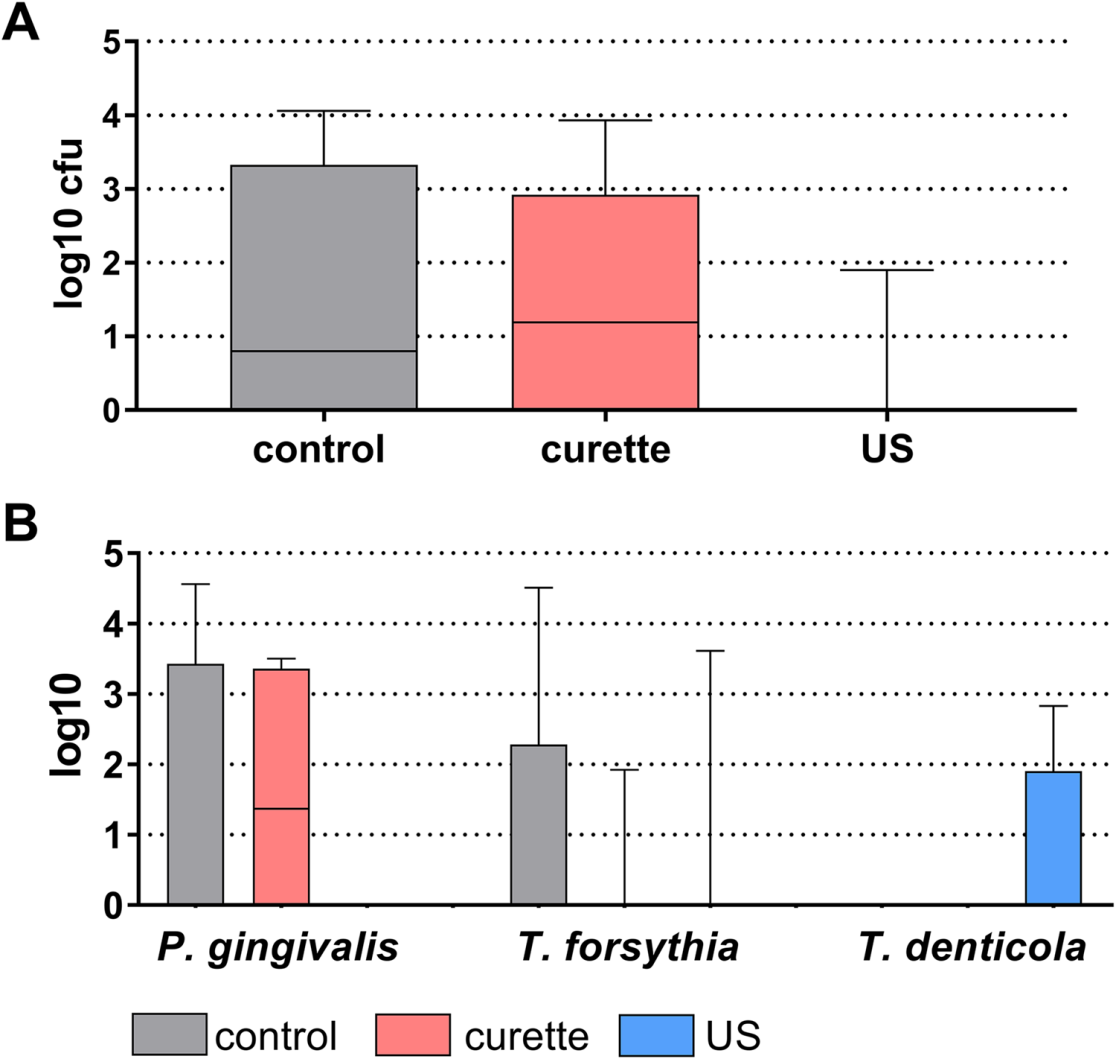
Median, quartiles, and range of total colony-forming units (cfu; **A**) and counts of selected bacterial species (determined by real-time PCR; **B**) adhering or invading epithelial cells 6 h after applying hand instrumentation (curette) and an ultrasonic scaler to biofilms on dentin discs

Four out of eight samples from the control and the curette group were tested positively for colony-forming units attaching or invading epithelial cells. *P. gingivalis* was detected in three controls and in four samples after instrumentation with curettes. Conversely, after ultrasonic instrumentation, only in one sample bacteria could be cultured. *P. gingivalis* was not identified at all, and *T. denticola*, which is not cultivable on regular blood agar plates, was detected in two samples. When comparing the counts of total cfu of selected bacteria, there was however no difference between the groups (Fig. 3A and B).

### IL-8 expression

**Fig. 4:**
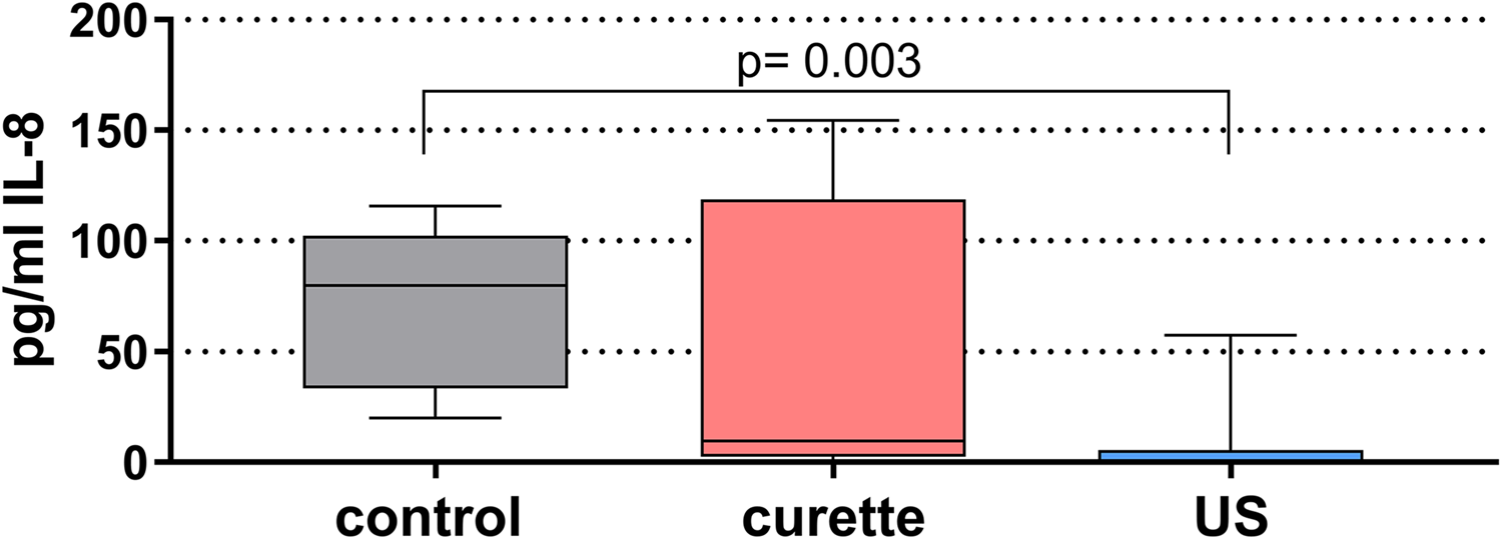
Interleukin-8 level (median, quartiles, and range) released from epithelial cells 6 h after applying hand instrumentation (curette) and an ultrasonic scaler to biofilms on dentin discs

IL-8 was quantified in the cell supernatants of epithelial cells 6 h after applying instrumentation to the biofilms. The difference between the three groups was statistically significant (*p* = 0.010). The followed up analysis found significantly lower IL-8 levels when a biofilm treated with US was exposed to epithelial cells in comparison with an untreated control (*p* = 0.003). Although the levels seemed to decrease after biofilm treatment with hand instruments in contact with epithelial cells, this difference failed statistical significance (Fig. 4).

## Discussion

In this study, we sought to develop a pocket model integrating epithelial cells and a multispecies pathogenic biofilm aiming to investigate the cell-biofilm interactions following treatment with two different (i.e., hand curettes or ultrasonic) instrumentation modalities.

A few in vitro studies analyzed the activity of therapeutics both on periodontal biofilm and on epithelial cells [22, 23, 32]; their purpose was to evaluate the anti-biofilm activity along with the therapeutics’ biocompatibility. Recently, a model has been developed to study the interaction of biofilm with epithelial cells when applying antiseptic natural oral health care products [25]. Similar to our model, biofilm was cultured first and independently then brought in close contact (without touching) to epithelial cells, and finally the test products were added to the system. In our model, however, it was impossible to apply instrumentation without touching and damaging the epithelial monolayer. Therefore, we decided to perform the instrumentation in the model first before adding the epithelial cells in order to not mechanically disturb the cells which might be considered as a limitation. All analyses were made 6 h after instrumentation when changes in the biofilm have already occurred: *T. denticola* was not detected in any of the controls; *P. gingivalis* was highly present in the biofilm composition, very often also after instrumentation. *Treponema* spp. are known for their extracellular location and close contact to epithelium [38]. Although it is well known that a biofilm consists of microorganisms and the surrounding biofilm matrix, we focused only on cfu analysis here. This might be related to the multitude of analyses to be performed at the same time. A more comprehensive biofilm analysis should be included in upcoming research.

The focus of the interaction between the biofilm after instrumentation and epithelial cells was put on the adhesion and invasion of bacteria and on the release of interleukin-8. Thereby, we did not differentiate between adhered and intracellularly located bacteria. Invasion of *P. gingivalis* and *T. forsythia* was studied several times [20, 28]; in the case of *P. gingivalis*, the activity of Arg-gingipains as a major virulence factor seems to be of importance [30]. As the method used for determining adhered incl. invasive bacteria destroys the epithelial cells, only released molecules could be used to assess the inflammatory response by the epithelial cells.

Given the inhibitory effect of *P. gingivalis* on IL-8 levels—it induces the degradation of IL-8 by gingipain proteases, a phenomenon called “local chemokine paralysis”—[6] a lower detection rate of *P.gingivalis* attached to or invaded into epithelial cells should result in higher IL-8 expression of the cells. However, this was not the case in our experiments. Epithelial cells exposed to discs after ultrasonic instrumentation exhibited a reduced IL-8 expression together with lower detection rates of *P*.*gingivalis* compared to control and hand instrumentation.

Considering that this is an in vitro model, it should be kept in mind that in an in vivo scenario the interplay of cells and biofilm depends on many more players on both sides with complex secretion patterns of virulence factors, endotoxins, and host cytokines. Moreover, the inflammatory status of the pocket epithelium along with irregularities on the root surface is likely to influence the treatment itself. Of course, in an in vitro situation, this complexity is lacking, and variances in host immune response or local site anatomy might disguise potential effects of each treatment approach. Also, standardized dentin discs were produced lacking any kind of irregularities. Nevertheless, this model mimicking a periodontal pocket provides an experimental setting to expose different instrumentation modalities, biofilm-inoculated dentin discs with epithelial cells. When thinking further along this path, a 3D model combining epithelial and other cell types (fibroblasts, monocytic cells) might be valuable.

Even though it has been well established that manual and ultrasonic instrumentation result in similar clinical outcomes in periodontitis patients [1, 17, 31], the two treatment modalities might influence the biofilm-cell interactions on a molecular level. Differences in biofilm removal 14 or water irrigation are likely to induce different cell responses of surrounding epithelial cells. The main findings of this study are that US instrumentation reduced the cfu counts significantly more than HI did together with less IL-8 release from epithelial cells compared to HI.

Our results showed that both treatment modalities substantially reduced bacterial counts, however, US to a greater extent than manual instrumentation. *P. gingivalis* was consistently found in nearly all samples. This is not surprising as *P. gingivalis* was proven to be hard to eliminate even after non-surgical mechanical therapy combined with systemic antibiotics [16, 27]. In our samples, *T. forsythia* was found in the majority of the control and to lower extent also in the test samples. Our detection rates agree with previously reported data by a clinical study. Within this context, *T. forsythia* was detected between 6.6 and 13.3% of sites in periodontitis patients after ultrasonic instrumentation [34]. In our study, *T. denticola* was found in only few samples of the control group and in even fewer of both test group samples. This is lining up with findings from a 2006 randomized clinical trial including patients diagnosed with former generalized aggressive periodontitis of whom subgingival samples were checked for periodonto-pathogenic bacteria using checkerboard DNA—DNA hybridization [36]. SRP alone resulted in a significant reduction of bacteria. *T. denticola* was identified at baseline in 81.8% of the patients and 6 weeks after SRP in 36.3% of the patients. This percentage was maintained up to the end of the follow-up time of 6 months. While these studies did not focus on hand versus ultrasonic instrumentation, Ioannou and co-workers found a significantly greater reduction of *T. forsythia* and *T. denticola* after 6 months favoring manual over ultrasonic instrumentation [14].

Next, we looked at the IL-8 expression of the epithelial cells. Here, US resulted in significantly lower IL-8 secretion by epithelial cells compared to the untreated control. IL-8 is a potent chemoattractant for neutrophils and responsible for neutrophil-induced tissue destruction [15]. It has been shown that IL-8 is elevated in periodontitis patients but is also being expressed in healthy controls. Its levels can be reduced by periodontal therapy [10]. In a clinical study, mechanical instrumentation resulted in a significant decrease of IL-8 levels in gingival crevicular fluid [21]. However, the interleukin levels did not correlate with the clinical outcomes. It has to be stressed out that instrumentation was performed using a combination of hand and ultrasonic instruments. In smokers with periodontal disease, non-surgical mechanical therapy (also here not further specified) induced a significantly higher increase of IL-8 compared to non-smokers with periodontal disease [33]. When comparing hand versus ultrasonic instrumentation in terms of inflammatory markers, no differences between IL-6, CRP, and TNF-α levels at days 1 and 7 after instrumentation were discerned [17]. Also, numerous in vitro studies showed that human gingival fibroblasts secrete increased levels of IL-8 in response to LPS from *P. gingivalis* [3, 13, 24]*.* As such, a recent study combined a three-dimensional gingival model with biofilms associated with both gingival health and gingivitis. They could show that cytotoxicity and IL-8 expression were rising with increasing maturity of the biofilm [4]. And yet, limited evidence exists on models integrating biofilm-cell interactions in different treatment modalities as here was done.

The clinical relevance of this in vitro study should be interpreted with caution. The transition from health to disease is accompanied by quantitative and qualitative alteration in the biofilm composition, and therefore, even the alteration in the commensal fraction of the biofilm could play a crucial part. However, a limitation of this study was that we concentrated on a few periodonto-pathogenic bacteria. Yet, our results imply that biofilm-cell interactions could be substantially influenced by different treatment modalities and in particular in view of the different IL-8 secretion might result in different tissue destruction. In the clinic, however, both manual and ultrasonic instrumentation are often combined making it difficult to see effects emerging from the treatment modality per se. This is also true for a vast number of studies, which often combined manual and ultrasonic instrumentation or missed to specify “SRP.” Our findings presented here leave many questions unanswered as for example to what extent water irrigation influenced the result or whether other cytokines relevant for periodontal inflammation or healing might be impacted. Further research should aim to establish well-suited models to further test the effects of different treatment modalities on cell-biofilm interactions and to increase the understanding of these complex interactions by including other cell types within periodontal pockets.
